# Inductive Effect of Palmatine on Apoptosis in RAW 264.7 Cells

**DOI:** 10.1155/2016/7262054

**Published:** 2016-05-31

**Authors:** Shintaro Ishikawa, Misako Tamaki, Yui Ogawa, Kiyomi Kaneki, Meng Zhang, Masataka Sunagawa, Tadashi Hisamitsu

**Affiliations:** Department of Physiology, School of Medicine, Showa University, 1-5-8 Hatanodai, Shinagawa-ku, Tokyo 142-8555, Japan

## Abstract

Osteoporosis is a serious public health problem characterized by low bone density and deterioration of the bone microarchitecture. Current treatment options target either osteoclast resorption or osteoblast formation. It has been reported that berberine, a close structural analog of palmatine, inhibited bone loss in an osteoporosis model. In this study, osseous metabolism was observed* in vitro* with osteoclast bone resorbing cells. We proved that mouse preosteoclastic cell line (RAW 264.7) has a higher sensitivity to palmatine than mouse osteoblastic cell line (MC3T3-E1); the cell survival rates significantly decreased at 40 *μ*M palmatine. The NO_2_^−^ level, a metabolic product of nitric monoxide (NO), and iNOS mRNA expression, an osteoclast with NO induced enzyme, also increased with higher dosage of palmatine. Furthermore, it was recognized that the cell viability decrease from palmatine was caused by apoptosis rather than necrosis. Additionally, osteoclast apoptosis from palmatine did not occur when iNOS was inhibited with N^G^-nitro-L-arginine methyl ester hydrochloride (pan NOS inhibitor). These results indicate that palmatine plays an important role in osteoclast apoptosis via the NOS system. Hence, palmatine could be considered as a viable pharmaceutical candidate for osteoporosis bone resorption inhibitor.

## 1. Introduction

Osteoporosis is a serious public health problem characterized by low bone density and deterioration of the bone microarchitecture. It results in a loss of bone strength and fractures. Nevertheless, bone is an active tissue that continues to remodel via bone formation and resorption. These two counteracting processes are strongly connected and firmly regulated to maintain skeletal homeostasis [1]. In other words, osseous metabolism depends on dual cell processes, osteoclasts and osteoblasts, differentiated bone resorbing cells derived from hematopoietic cells of monocyte-macrophage lineage, and bone forming cells of mesenchymal origin, respectively. Bone diseases frequently occur because of differentiation aberration or proliferation activity in the osteoclast and osteoblast. Current treatments for osteoporosis target either the osteoclast by inhibiting bone resorption (antiresorptive agents) or the osteoblast by stimulating bone formation (osteoanabolic agents).

Bone resorption of postmenopausal women progresses approximately two times faster than bone formation [2]. The possibility of suffering a fracture increases in women with decreased bone quantity, even if osteoporosis is in its early stages [3]. When bone quantity decreases (early stage), a physician may give advice on lifestyle improvement to the patient (e.g., diet and exercise). As osteoporosis progresses, pharmacotherapy, which inhibits bone resorption, will be an important treatment option. The first choice is usually bisphosphonate or raloxifene. An estrogenic pharmaceutical is chosen when patients have menopausal syndrome. However, careful medication selection will be crucial because of its possible side effects.

Palmatine is a quaternary protoberberine alkaloid. It is typically yellow in color and is an active constituent in a number of plants, such as* Rhizoma Coptidis* [4]. Alkaloids have been used in the treatment of jaundice, dysentery, hypertension, inflammation, and liver-related diseases [5]. It was previously reported that berberine, an isoquinoline alkaloid, which is a close structural analog of palmatine, inhibited bone loss in an osteoporosis model [6].

Our findings showed evidence that palmatine regulated osteoclast activity by secretion of cytokines of osteoblasts [7], but we did not discuss palmatine's influence on osteoclast-induced bone resorption. Because the effect of resorption could prevent osteoclast activity or directly inhibit osteoclasts, we investigated the influence of palmatine on osteoclast differentiation and function.

## 2. Materials and Methods

### 2.1. Reagents and Cell Culture

Palmatine chloride (Wako Pure Chemical Industries, Ltd., Tokyo, Japan) was used in the present study. Palmatine was dissolved in a culture medium in* in vitro* experiments. The culture medium used was Dulbecco's modified eagle medium (DMEM; Sigma-Aldrich Corporation, St. Louis, MO, USA) or alpha modified eagle minimum essential medium (*α*-MEM; Sigma-Aldrich Co.) supplemented with 10% heat-inactivated fetal calf serum (FCS; Nihon Bio-Supply Center, Tokyo, Japan) and a penicillin-streptomycin-neomycin (PSN) antibiotic mixture (5 mg each of penicillin and streptomycin and 10 mg of neomycin/mL, GIBCO 15640, Life Technologies, Inc.), sterilized with 0.2 *μ*m pore filters, and stored at 4°C until its use. The mouse osteoblastic cell line MC3T3-E1 (DS Pharma Biomedical Co., Ltd., Osaka, Japan) was routinely cultured at 37°C in a humidified atmosphere of 5% CO_2_ and maintained in *α*-MEM-FCS-PSN. The transformed murine monocytic cell line RAW 264.7 (DS Pharma Biomedical Co., Ltd.) was routinely cultured at 37°C in a humidified atmosphere of 5% CO_2_ and maintained in DMEM-FCS-PSN. N^G^-Nitro-L-arginine methyl ester hydrochloride (L-NAME) was obtained from Dojindo Laboratories, Kumamoto, Japan.

### 2.2. Cell Viability in Cytotoxicity CCK-8 Assays

Cell viability was measured using the cell counting kit-8 (CCK-8; Dojindo, Kumamoto, Japan) [8]. The cells were seeded in a 96-well flat-bottomed microplate (5 × 10^3^ cells/well) and cultured in 100 *μ*L of growth medium at 37°C and 5% CO_2_ for 24 h or 5 days. The cell culture medium in each well was then replaced with 100 *μ*L of cell growth medium containing palmatine at the following concentrations: 1, 5, 10, 40, and 100 *µ*M [9, 10]. Additionally, L-NAME (10, 100, and 1000 *μ*M) dissolved in PBS [11] was added. After incubation for 24 h or 5 days at 37°C, the cells were washed with PBS 3 times. Then, 10 *μ*L of CCK-8 dye and 100 *μ*L of *α*-MEM cell culture medium were added to each well, and cells were incubated for another 1 h at 37°C. Cell viability (%) was calculated using the following formula:

(1) Cell  viability%=ODexperimental  sample−ODblankODcontrol−ODblank×100%.

OD_experimental  sample_  refers to the absorbance of a well with treated cells and CCK-8. OD_blank_ refers to the absorbance of a well with medium and CCK-8 but without cells. OD_control_  refers to the absorbance of a well with untreated cells and CCK-8 [7, 8]. The absorbance at 450 nm was measured by a microplate reader (Multiskan*™* GO instrument; Thermo Fisher Scientific Inc., Waltham, MA, USA), and the results are presented as mean ± SD from triplicate wells.

### 2.3. Establishment of a Coculture System for Bone Resorption

It has been known that osteoblasts and osteoclasts interact in bone tissue [12]. Therefore, we observed the influence of palmatine for osteoblast and osteoclast crosstalk under a coculture in an* in vitro* system.

A novel coculture system was established using Transwell inserts (Corning Incorporated Number 3450, NY, USA) [12]. Osteoclast bone resorption activity was assessed using a bone resorption assay kit 24 (PG Research, Tokyo, Japan), under the same culture conditions as described above [13]. The bottom of the inserts was composed of polyester materials with a pore size of 0.4 *µ*m, which only permits the passage of small, soluble factors. RAW 264.7 cells, a mouse preosteoclastic cell line, were embedded at a density of 5 × 10^5^ cells/cm^2^ in the lower compartment, bone resorption assay plate, of each insert. MC3T3-E1 cells, a monoclonal preosteoblastic cell line, were incubated at a density of 5 × 10^4^ cells/cm^2^ in the upper compartment of the inserts. After each cell was cultured for 24 h, the Transwell inserts with MC3T3-E1 cells were combined together in the bone resorption assay plate with RAW 264.7 cells. This coculture system was maintained in DMEM-FCS-PSN and supplemented with different concentrations of palmatine in 5% CO_2_ at 37°C. After coculturing for 5 days, the sequential experiments were performed.

### 2.4. Quantification of Osteoclastic Activity: Biomimetic Calcium Phosphate Assay and Resorption Pit Assay

The cocultured cells were incubated on bone resorption assay plates and fluorescein-labeled CaP-coated 24-well plates without medium change under the light-shielded condition. After 5 days, 100 *μ*L of the cell culture supernatant was transferred into a 96-well plate for fluorescence measurement, mixed with 50 *μ*L of bone resorption assay buffer added to each well, and then mixed using a plate shaker. The fluorescence intensity, fluoresceinamine-labeled chondroitin sulfate, was measured using a fluorescence plate reader (Twinkle LB970, Berthold Japan, Tokyo, Japan) with excitation and emission wavelengths of 485 and 535 nm. The remaining plates were washed with PBS and treated with 5% sodium hypochlorite for 5 min. After washing the plates with tap water and drying them, five different regions in each well were photographed by microscopy (Olympus Co.) and the pit areas were measured with ImageJ software v. 1.48 (NIH, USA) [14].

### 2.5. Coculture for Tartrate-Resistant Acid Phosphatase (TRAP) Stain

RAW 264.7 cells and MC3T3-E1 cells were mixed at a rate of 5 × 10^4^ cells and disseminated in the 24-well plate (IWAKI 3820-024; Asahi Glass Co., Ltd., Tokyo, Japan). This coculture system was maintained in DMEM-FCS-PSN and supplemented with different concentrations of palmatine in 5% CO_2_ at 37°C. After coculturing for 5 days, the sequential experiments were performed. To confirm the generation of multinucleated osteoclast-like cells, the cultured RAW 264.7 cells were stained with TRAP (TRAP/ALP stain kit, Wako Pure), according to the manufacturer's instructions. TRAP-positive multinucleated (3 or more nuclei) osteoclasts were visualized by light microscopy and photographed. The number of mature osteoclasts, multinucleated RAW 264.7 cell, per a field (quadrangle of 2 mm × 2.5 mm) was counted to quantify the influence of palmatine [15, 16]. Each osteoclast formation assay was performed at least 3 times.

### 2.6. Apoptosis Detection

RAW 264.7 cells were plated in 24-well tissue culture plates at a cell density of 1 × 10^5^ cells per well. After 24 h, the medium was replaced with medium containing the palmatine compounds. All treatments were performed in triplicate. The cells were treated for 24 h, after which they were harvested and the extent of apoptosis was assessed using the APOPercentage*™* assay (Biocolor Ltd., Newtownabbey, Northern Ireland, UK) as previously described [17]. Briefly, the cells were removed by trypsinization, washed with PBS, and stained with APOPercentage dye for 30 min at 37°C. The dye uptake was quantified by the colorimetric method based on the manufacturer's instructions. The cells were lysed with the dye release reagent of attachment, and the absorbance was measured at 550 nm using a Multiskan GO instrument.

### 2.7. Detection of Supernatant Nitric Monoxide Level

The amount of nitrite/nitrate in the supernatant produced by RAW 264.7 cells was measured using an NO_2_/NO_3_ assay kit FX (NK08; Dojindo Laboratories, Kumamoto, Japan), based on manufacturer's instructions. The cells were seeded in a 24-well flat-bottomed microplate (1 × 10^5^ cells/well) and cultured in 1000 *μ*L of DMEM-FCS-PSN medium at 37°C and 5% CO_2_ for 24 h. The cell culture medium in each well was then replaced with 1000 *μ*L of cell growth medium containing palmatine at the following concentrations: 1, 5, 10, 40, and 100 *µ*M [9, 10]. Then, L-NAME (10, 100, and 1000 *μ*M), dissolved in PBS [11], was added. After incubation for 2 h, 4 h, or 5 days at 37°C, 80 *µ*L of the supernatant was transferred to an empty 96-well plate, 2,3-diaminonaphthalene reagents of attachment were added to each well, and the plate was incubated. Fluorescence intensity was measured using a Twinkle LB970, with excitation and emission wavelengths of 355 and 450 nm.

### 2.8. PCR Primers and Reagent Kits

The reagents used for mRNA isolation (TaqMan Gene Expression Cells-to-CT*™*) and real-time reverse transcription-polymerase chain reaction (RT-PCR; TaqMan Gene Expression Assays) were purchased from Applied Biosystems (Foster City, CA, USA). Assays were conducted according to the manufacturer's instructions [18]. For RT-PCR comparison of gene expression, we selected* iNOS* (NOS2: TaqMan Gene Expression Assays; Assay ID: Mm00440502_m1). The 18S ribosomal RNA (Mm18s: TaqMan Gene Expression Assays; Assay ID: Mm03928990_g1) was used as a housekeeping gene to normalize RNA loading.

### 2.9. mRNA Isolation and Quantitative RT-PCR

After incubation for 2 h at 37°C, total RNA was isolated from RAW 264.7 cells using 50 *μ*L of a lysis solution (P/N4383583). Each sample of total RNA was subjected to RT using a 20 × RT enzyme mix (P/N 4383585) and a 2 × RT buffer (P/N43833586) with a T100 thermal cycler (Bio-Rad Co., Hercules, CA, USA). After RT reaction, the cDNA templates were amplified by PCR using TaqMan Gene Expression Assays, PCR primers, and RT master mix (P/N 4369016). Predesigned and validated gene-specific TaqMan Gene Expression Assays [17–19] were duplicated for quantitative RT-PCR, based on manufacturer's protocol. PCR assays were conducted as follows: 10 mins of denaturation at 95°C, 40 cycles of 15 s, denaturation at 95°C, and 1 min annealing and extension at 60°C. Samples were analyzed using an ABI Prism 7900HT Fast RT-PCR System (Applied Biosystems) [19, 20]. Relative quantification (RQ) studies [21] were prepared from collected data (threshold cycle numbers (*Ct*)) with ABI Prism 7900HT Sequence-Detection System (SDS) software v. 2.3 (Applied Biosystem).

### 2.10. Statistical Analysis

Data is expressed as means ± SD. All assays were repeated 3 times to ensure reproducibility. Statistical significance between the control and experimental groups was analyzed by one-way analysis of variance followed by Scheffé test. Probability (*p*) value < 0.05 was considered statistically significant.

## 3. Results

### 3.1. TRAP-Positive Cell Detection on Coculture

**Figure 1:**
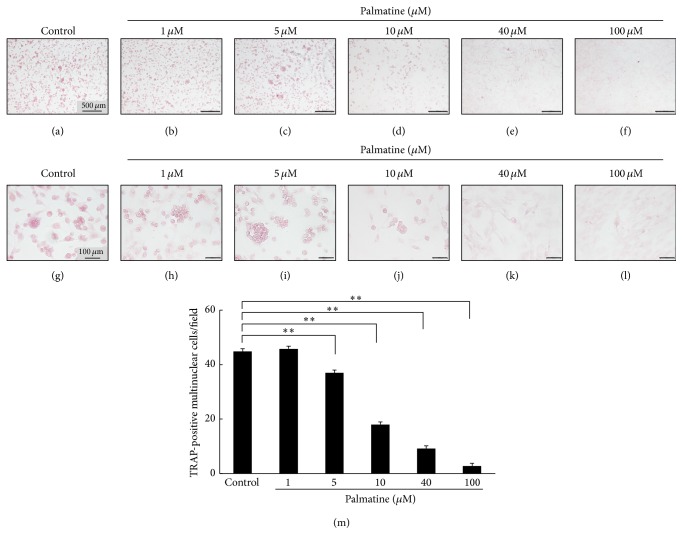
TRAP-positive cell detection with the coculture method. The differentiation to osteoclast of a RAW 264.7 cell was examined using TRAP staining in culture of a RAW 264.7 cell and an MC3T3-E1 cell for 5 days. Digital images were obtained using an optical microscope. RAW 264.7 cells, which were dyed using TRAP in the cell block section, show differentiation of a RAW 264.7 cell, cytogamy image. (a)–(f) ×100 magnification, (g)–(l) ×600 magnification. (m) The number of mature osteoclasts, multinucleated RAW 264.7 cell, per a field (quadrangle of 2 mm × 2.5 mm) was counted to quantify influence of palmatine. Asterisks indicate statistically significant differences (^*∗∗*^ *p* < 0.01). Error bars denote ±SD.

It is known that RAW 264.7 cells differentiate into osteoclastic cells over days in culture [22]. Therefore, RAW 264.7 cells' differentiation to osteoclast was examined by TRAP staining in culture of RAW 264.7 cells and MC3T3-E1 cells. As shown in Figure 1, RAW 264.7 cell fusion began and multinucleated giant cells developed in the control group on culture day 5. The multinuclear osteoclast per a field (quadrangle of 2 mm × 2.5 mm) decreased according to palmatine's dosage (Figure 1(m)).

### 3.2. Cell Viability for Palmatine with Cytotoxicity CCK-8 Assays

**Figure 2:**
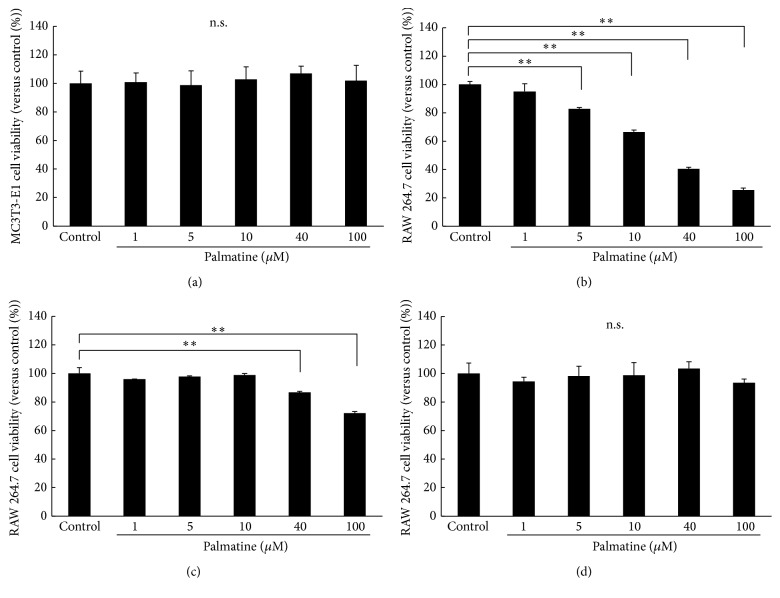
Cell viability for palmatine with cytotoxicity CCK-8 assays. The proliferation of cells cultured with different palmatine concentrations for 24 h or 5 days was evaluated using a CCK-8 assay. Seeded cells were incubated with or without palmatine for 24 h or 5 days. The cell viability was calculated using the following formula: cell viability (%) = [OD_experiment_ − OD_blank_]/[OD_control_ − OD_blank_] × 100%. The absorbance was measured at 450 nm. (a) The survival rate of MC3T3-E1 cells that were incubated in palmatine component medium for 5 days. (b) The survival rate of RAW 264.7 cells that were incubated in palmatine component medium for 5 days. (c) The survival rate of RAW 264.7 cells that were incubated in palmatine component medium for 24 h. (d) The survival rate of mature RAW 264.7 cells that were incubated in palmatine component medium for 5 days. Asterisks indicate statistically significant differences (^*∗∗*^ *p* < 0.01, n.s., no significant difference). Error bars denote ± SD.

The proliferation of cells cultured with different palmatine concentrations was evaluated using the CCK-8 assay. Five days of palmatine treatment even at high dosage of 100 *µ*M (Figure 2(a)) did not change the MC3T3-E1 cell survival rates. However, at concentration >5 *µ*M (Figure 2(b)), RAW 264.7 cell survival rate significantly decreased.

Palmatine treatment of 24 h at concentration >40 *µ*M (Figure 2(c)) significantly decreased the RAW 264.7 cell survival rate. Furthermore, the survival rate of the osteoclast-like differentiated mature RAW 264.7 cells, which were cocultured with MC3T3-E1, was not affected even by the high dosage of 100 *μ*M palmatine (Figure 2(d)).

### 3.3. Apoptosis Detection with APOPercentage Assay

**Figure 3:**
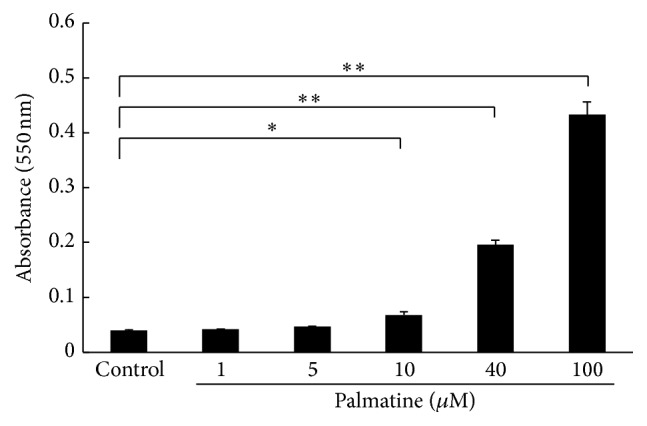
Apoptosis detection with an APOPercentage assay. The cell apoptotic rate was measured using an APOPercentage assay after the seeded cells were incubated with each palmatine concentration for 24 h. The absorbance at 550 nm was measured. Asterisks indicate statistically significant differences (^*∗*^ *p* < 0.05, ^*∗∗*^ *p* < 0.01). Error bars denote ± SD.

An apoptosis assay was carried out to confirm whether the cellular extinction by palmatine depends on necrosis or apoptosis. The pigmentary absorbance which was extracted from the apoptosis cell was measured. As shown in Figure 3, palmatine treatment for 24 h at concentration >10 *µ*M significantly increased RAW 264.7 cell apoptosis rate.

### 3.4. Supernatant NO_2_^−^ Level Detection

**Figure 4:**
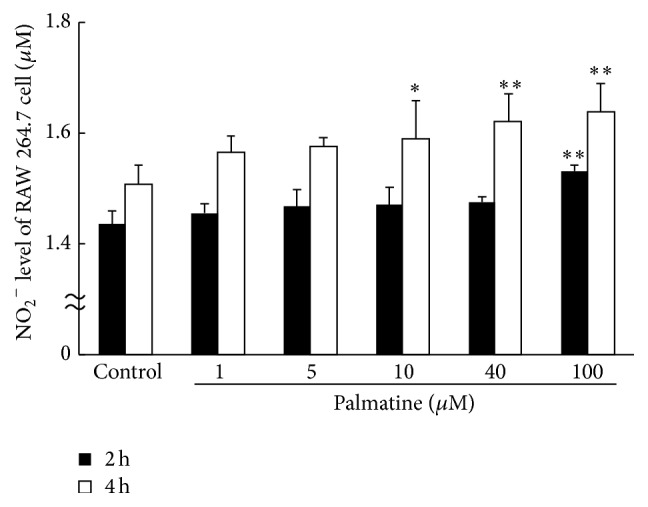
Supernatant NO_2_^−^ level as a surrogate for NO. Nitrous acid ion (NO_2_^−^) production in RAW 264.7 cells was measured using the NO_2_/NO_3_ assay kit FX. The black bars show the supernatant NO_2_^−^ level that cultured RAW 264.7 cells for 2 h. The white bars show the supernatant NO_2_^−^ level which cultured RAW 264.7 cells for 4 h. Asterisks indicate statistically significant differences (^*∗*^ *p* < 0.05, ^*∗∗*^ *p* < 0.01 versus control). Error bars denote ± SD.

An experiment was performed to determine whether palmatine affected nitrous acid ion (NO_2_^−^) production in RAW 264.7 cells using the NO_2_/NO_3_ assay kit FX. As shown in Figure 4, palmatine treatment of 2 h at high concentration 100 *µ*M significantly increased the NO production in RAW 264.7 cells. Furthermore, a treatment of 4 h significantly increased the NO production in accordance with palmatine concentration.

### 3.5. iNOS mRNA Appearance in RAW 264.7 Cell

**Figure 5:**
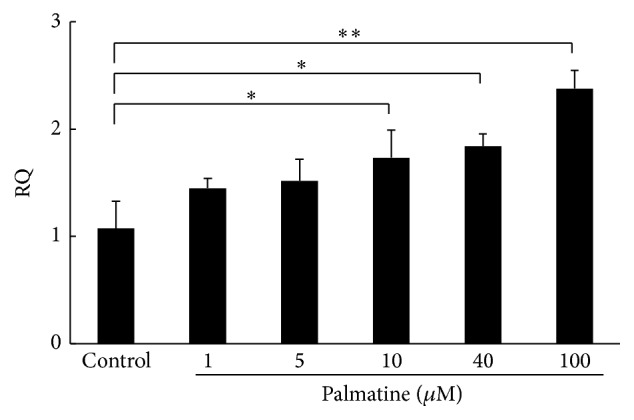
Relative quantity (RQ) of iNOS mRNA expression in RAW 264.7 cells. The experiment was conducted to examine whether addition of palmatine would change the iNOS mRNA expression in RAW 264.7 cells. After culture for 2 h, RAW 264.7 cells were collected and used for the measurement of iNOS mRNA expression by RT-PCR. Asterisks indicate statistically significant differences (^*∗*^ *p* < 0.05, ^*∗∗*^ *p* < 0.01). Error bars denote ± SD.

The next experiment examined whether addition of palmatine could change iNOS mRNA expression in RAW 264.7 cells using RT-PCR method. The proliferation of cells cultured with different palmatine dosages was examined using the CCK-8 assay. As shown in Figure 5, palmatine treatment of 2 h at concentration >10 *µ*M significantly increased the iNOS mRNA expression in RAW 264.7 cells.

### 3.6. Cell Viability and Supernatant NO_2_^−^ Level Detection for L-NAME Treatment

**Figure 6:**
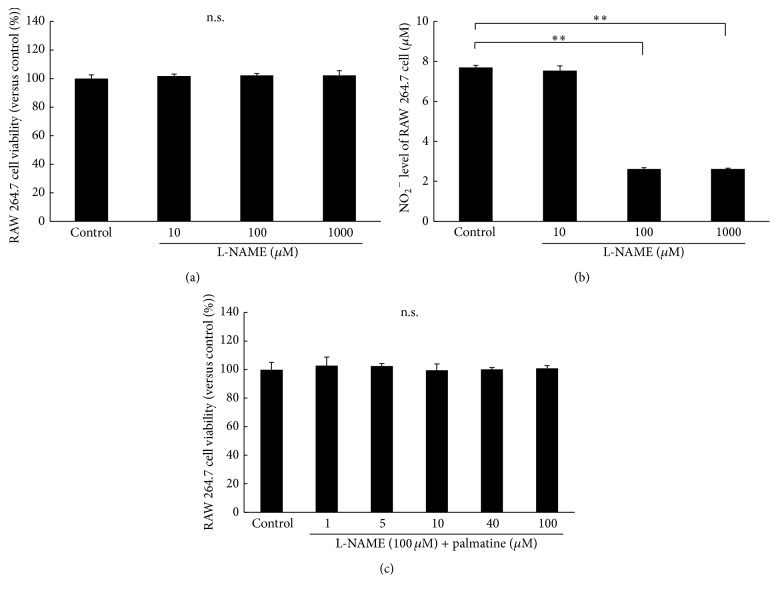
Cell viability and supernatant NO_2_^−^ level detection for L-NAME treatment. NO release was inhibited by the addition of L-NAME to clarify whether NO affects the survival of RAW 264.7 cells. ((a) and (b)) The cell survival rate after culture and NO_2_^−^ level were examined to clarify the direct influence of L-NAME on RAW 264.7 cells after 5 days in culture medium containing L-NAME. (c) The proliferation of cells cultured with different palmatine concentrations for 24 h was evaluated using a CCK-8 assay. The cell viability was calculated using the following formula: cell viability (%) = [OD_experiment_ − OD_blank_]/[OD_control_ − OD_blank_] × 100%. Absorbance was measured at 450 nm. Nitrous acid ion (NO_2_^−^) production in RAW 264.7 cells was measured using the NO_2_/NO_3_ assay kit FX. Asterisks indicate statistically significant differences (^*∗∗*^ *p* < 0.01, n.s., no significant difference). Error bars denote ± SD.

In order to assess whether NO affects the survival of RAW 264.7 cells, L-NAME was added. Firstly, to clarify the direct effect of L-NAME on the survival of RAW 264.7 cells, they were both cocultured for 5 days. Then the cell's condition of the controlled group and NO_2_^−^ level were examined. The result was that the NO_2_^−^ level decreased significantly, by 100 *μ*M. The survival rate of the cells did not have significant difference in each of the concentrations. Therefore, the optimum concentration level of L-NAME, which has no cytotoxic effects and inhibits NO secretion, was set at 100 *μ*M. L-NAME was dissolved in PBS and added to the culture medium (final concentration: 100 *μ*M). The proliferation of cells cultured with different palmatine concentrations level was evaluated using the CCK-8 assay. As shown in Figure 6(c), L-NAME treatment of 24 h did not change RAW 264.7 cell survival rates at any concentrations level.

### 3.7. Bone Resorption Evaluation on Coculture

**Figure 7:**
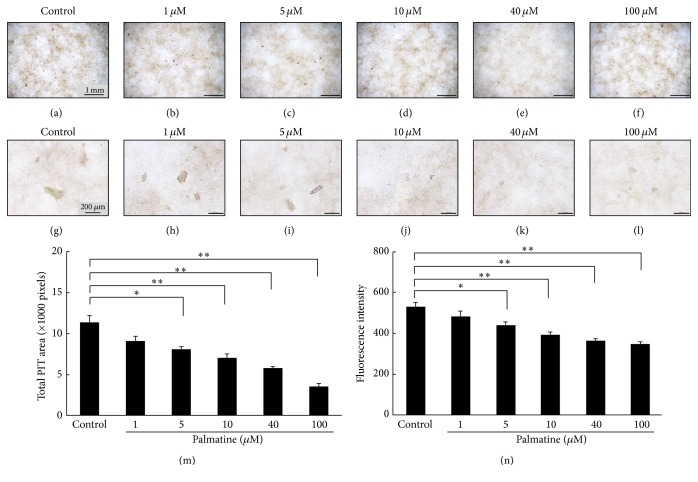
Bone resorption evaluation with a CaP-coated plate. Bone resorption ability of a RAW 264.7 cell examined using a bone resorption assay kit in culture of a RAW 264.7 cell and an MC3T3-E1 cell for 5 days. The bone resorption activity was evaluated by measuring the pit formation on a CaP-coated plate and the fluorescence intensity of the conditioned medium. (a)–(l) Digital images of pit formation on a CaP-coated plate were obtained using an optical microscope. The brown pit shows the locus where a mature RAW 264.7 cell (differentiation like osteoclast) dissolved a calcium plate. (a)–(f): ×40 magnification, (g)–(l): ×400 magnification. (m) The total pit area on a CaP-coated plate. (n) The fluorescence intensity of bone resorption dependence in the conditioned medium. Asterisks indicate statistically significant differences (^*∗*^ *p* < 0.05, ^*∗∗*^ *p* < 0.01). Error bars denote ± SD.

Bone resorption ability of RAW 264.7 cells was examined using a bone resorption assay kit in the culture of RAW 264.7 and MC3T3-E1 cells. Bone resorption activity was evaluated by measuring the pit formation on a CaP-coated plate and the fluorescence intensity of the conditioned medium. The microscopic photographs (Figures 7(a)–7(l)) and the total pit area (Figure 7(m)) indicated that the pit formation decreased with increasing palmatine dosage. The fluorescence intensity of the conditioned medium also significantly decreased with increasing palmatine dosage (Figure 7(n)). These assay evaluations showed a similar tendency.

## 4. Discussion

Bone is a living tissue that is constantly being degraded and replaced. Osteoporosis occurs when the creation of new bone does not keep up with the removal of old bone. In the current study, we examined the influence of palmatine on osteoclast function using RAW 264.7 cells and MC3T3-E1 cells* in vitro*.

The first part of this study was undertaken to examine the effects of palmatine on the differentiation on RAW 264.7 cells. Results of a coculture with MC3T3-E1, osteoblast-like cell, and RAW 264.7, osteoclast-like cell, and the osteoclast differentiation on TRAP staining were inhibited by palmatine dosage (Figure 1). The preliminary research showed that palmatine affected the bone immunological function in osteoblast and osteoclast. However, palmatine's effects on osteoclasts were not concluded.

Therefore, the second experiment in this study examined the direct influence of palmatine on osteoclast survival. Our results proved that RAW 264.7 cells have a higher sensitivity to palmatine than MC3T3-E1 cells. The survival rate significantly decreased as palmatine's dosage and exposure period increased (Figure 2). Furthermore, palmatine did not alter the cocultured, osteoclast differentiated mature RAW 264.7 cell. In other words, the result indicated that palmatine may be related to primary osteoclast differentiation or proapoptotic activity. Additionally, an apoptosis assay was carried out to confirm whether the cellular extinction from palmatine depends on necrosis or apoptosis. Apoptosis is a multistage process in which activity of caspase enzymes fluctuates, DNA becomes fragmented, and phosphatidyl serine is transferred to the exterior surface of the cell membrane [23]. Such transfer and exposure of phosphatidyl serine are linked to the onset of apoptosis. Necrotic cells are not involved in this transfer. Therefore, the degree of apoptosis was determined by this phosphatidyl serine transmembrane movement. We identified that the decrease in dosage of palmatine reduces RAW 264.7 cells and thereby induces apoptosis, rather than necrosis. Additionally, apoptosis increases according to the dosage of palmatine in a concentration-dependent manner (Figure 3). Further, it has been known that nitric oxide induces apoptosis in various cells lines. Simultaneously, activating the NO/cGMP signaling pathway, including NO, prevents apoptosis induced by diverse stimuli [24]. Even if higher density was applied to the MC3T3-E1 cells, apoptosis did not occur [7]. Thus, experimenting over a range of densities with palmatine may activate the NO/cGMP signal in the MC3T3-E1 cells.

The purpose of the third experiment was to find the factor that induced osteoclast apoptosis. Some researchers previously reported that NO was the important factor which induced osteoclast apoptosis [25–27]. The mechanism that NO induces apoptosis in a macrophage-like cell is related to the release of cytochrome c from mitochondria, increase in the expression of the tumor suppressor p53, and accumulation of proapoptotic factors (e.g., the Bcl-2 family) [28]. Therefore, we investigated the relationship of palmatine and NO. The level of NO_2_^−^, a metabolic product of NO, was measured since half-life of NO is very short (3–6 s). NO in the culture supernatant increased as palmatine concentrations increased (Figure 4). Furthermore, the iNOS mRNA expression, which was an NO induced enzyme in osteoclast, also increased as palmatine concentrations increased (Figure 5). Additionally, osteoclast apoptosis from palmatine did not occur when iNOS was previously inhibited with L-NAME (pan NOS inhibitor/Figure 6). Furthermore, as palmatine dosage increased, bone resorption decreased (Figure 7). These results suggest that palmatine inhibits bone resorption and osteoclast formation, which supports our preliminary research [7]. It has also been reported that NO inhibits osteoclast differentiation [29] and bone resorption [30]. Hence, palmatine not only induces osteoclast apoptosis but also may inhibit osteoclast activities.

These results provide the first evidence that palmatine plays an important role in osteoclast apoptosis via the NOS system in osteoclasts. Thus, palmatine could be considered as a viable pharmaceutical candidate for osteoporosis bone resorption inhibitor. NO has been reported to be a mediator of bone turnover that has biphasic effects on regulating osteoblast survival and death [31]. NO may affect normal bone resorption by osteoclasts. Additionally, RAW 264.7 is a leukemic cell line. The apoptotic reactions of cancer cell lines to drug treatment may be different from normal monocytic cells. Therefore, we believe that palmatine's examination under varied conditions and on the nuclear intrinsic factor of normal osteoclasts would be necessary, as long as the role of NO on osteoclast activity is yet to be determined.
